# Prevalence of Psychopathy in the General Adult Population: A Systematic Review and Meta-Analysis

**DOI:** 10.3389/fpsyg.2021.661044

**Published:** 2021-08-05

**Authors:** Ana Sanz-García, Clara Gesteira, Jesús Sanz, María Paz García-Vera

**Affiliations:** Departamento de Personalidad, Evaluación y Psicología Clínica, Universidad Complutense de Madrid, Madrid, Spain

**Keywords:** psychopathy, prevalence, general population, meta-analysis, PCL-R

## Abstract

The main objective of this study was to systematically and meta-analytically review the scientific literature on the prevalence of psychopathy in the general adult population. A search in PsycInfo, MEDLINE, and PSICODOC identified 15 studies published as of June 2021. Altogether, 16 samples of adults totaling 11,497 people were evaluated. Joint prevalence rates were calculated using reverse variance heterogeneity models. Meta-regression analyses were conducted to examine whether the type of instrument, sex, type of sample, and country influenced prevalence. The meta-analytical results obtained allow us to estimate the prevalence rate of psychopathy in the general adult population at 4.5%. That being said, this rate varies depending on the participants' sex (higher in males), the type of sample from the general population (higher in samples from organizations than in community samples or university students), and the type of instrument used to define psychopathy. In fact, using the PCL-R, which is currently considered the “gold standard” for the assessment and definition of psychopathy, the prevalence is only 1.2%. These results are discussed in the context of the different theoretical perspectives and the existing problems when it comes to defining the construct of psychopathy.

## Introduction

The construct of psychopathy is understood generically as a type of personality disorder characterized, among other important features, by the presence of behaviors that conflict with the social, moral, or legal norms of society, giving rise in many cases to clearly criminal behaviors (Hare, 2003a,b; Crego and Widiger, 2018; Lykken, 2018; Patrick, 2018; Widiger and Crego, 2018). This construct is widespread in the area of psychopathology or personality psychology, but above all, in the area of legal and forensic psychology, including criminal and prison psychology. In fact, for some researchers, such as Hare (1998), psychopathy is one of the most important clinical constructs in these areas. This is because the personality and behavior of offenders with a diagnosis of psychopathy are very different from those of other offenders. Furthermore, according to Hare (1998), those differences are not only as important as environmental, social, and situational factors to understand crime, but they also allow us to improve the assessment of the risk of recidivism and violence and select appropriate treatment programs.

However, the concept of psychopathy has evolved throughout history and in this evolution, perhaps a key moment for the objectives of the present work is the proposal made by Hervey Cleckley in the 1940s (Cleckley, 1988). Cleckley proposed a very clear distinction between the criminals and the person suffering from psychopathy. What fundamentally defines psychopathy is not criminal behavior, but the presence of a series of personality traits, generally related to a lack of emotion (e.g., lack of nervousness, absence of remorse or shame, inability to love, shallow affective reactions) and the presence of an outward appearance of normality (e.g., lack of delusions and other signs of irrational thought, superficial charm, and good “intelligence”). Therefore, for Cleckley, not all criminals are psychopaths, and not all psychopaths are criminals. Hence, there would be people in the general population who would have a psychopathic personality, but who would never have committed any crime and will perhaps never commit one. However, they will manifest socially maladaptive and ethically reprehensible behaviors (e.g., failure to follow a life plan, impersonal, trivial, and poorly integrated sex life, deceitfulness and lack of sincerity, pathological egocentrism).

As he (Hare, 2013) acknowledged, Cleckley's conception of psychopathy greatly influenced the work of Robert Hare's. Hare has become one of the highest authorities in the field of psychopathy, especially since the elaboration and publication in 1991 of the Hare Psychopathy Checklist-Revised or PCL-R (Hare, 1991). The PCL-R has, over the years, become the “gold standard” for the evaluation of psychopathy in forensic and prison contexts (Edens et al., 2001).

The PCL-R is a symptom construct rating scale of 20 items that, using a semi-structured interview and data from the case files and other collateral information, provides a score in psychopathy that can range from 0 to 40. The cut-off score of 30 (30 or more) is the most used to classify a person as a psychopath. However, some research has used a cut-off score of 25 to identify subclinical psychopathy, which already indicates a high level of psychopathy (Hare, 2013).

The items of the PCL-R are intended to cover most of Cleckley's characteristics of psychopathy. However, as the instrument was developed and validated in the prison population, the concept of psychopathy underlying the PCL-R attaches more weight to the characteristics of criminality and a socially deviated lifestyle than Cleckley's original conception. This difference is reflected in PCL-R items such as juvenile delinquency, parole revocation, and criminal versatility (Items 18, 19, and 20, respectively).

The application and scoring of the PCL-R requires a lot of time and involves access to information from files and collateral sources. Therefore, in 1995, an abbreviated version was developed for its use in screening task: the Hare Psychopathy Checklist-Screening Version or PCL:SV (Hart et al., 1995). This version has only 12 items and does not require consultation of other sources of information. When using the PCL:SV in forensic and prison population, it is common to use a cut-off score of 18 (or more) to identify psychopathy and a cut-off score of 13 (or more) to identify subclinical psychopathy.

Drawing on the PCL-R and its different versions, including the PCL:SV, the scientific literature on psychopathy in the forensic or prison population has grown dramatically in the last 30 years. Hence, there are currently many correlational studies, but also experimental or laboratory studies that have addressed very different aspects of this construct in this type of population. These aspects are its structure, etiology, psychological mechanisms and psychobiological correlations to its relationship with a wide variety of factors (e.g., sex, age, socioeconomic status, education, family environment, ethnicity, culture). Also, its ability to predict diverse socially or clinically relevant behaviors (e.g., violence, criminal recidivism, gender-based violence, sexual offending, institutional misconduct, alcohol and substance abuse, response to treatment) (see reviews of Leistico et al., 2008; Hare, 2013; Fox and DeLisi, 2019).

The results of some of these investigations that have examined differences in psychopathy based on sex or culture are particularly relevant to the aims of this study. Research has consistently found that the prevalence of psychopathy is higher in male offenders and prisoners than in female offenders and prisoners (see the review of Beryl et al., 2014). It has also been found that psychopathy prevalence and psychopathy levels are higher in North American male and female prisoners than in European male and female prisoners (see the reviews of Beryl et al., 2014; Fox and DeLisi, 2019).

Despite all the data that support the validity and usefulness of the psychopathy construct, a source of controversy concerning it has to do with the possibility of considering it as a categorical or a dimensional construct. A categorical classification allows a clear differentiation between people who have psychopathy and people who don't, because there would be qualitative differences between them. In contrast, if it is considered as a dimensional construct and therefore, a maladaptive variant of the normal personality, there would only be quantitative differences between such persons.

The latter possibility is supported by a growing scientific literature concerning at least four major lines of research and argumentation: (1) the relationship between psychopathy and the five-factor personality model, also known as the Big Five model, and which is currently considered the most validated and consensual model of personality traits (e.g., Widiger and Lynam, 1998; Deferinko and Lynam, 2013; Vachon et al., 2013; Lynam and Miller, 2019); (2) the relationships between the PCL-R and other instruments specifically designed to measure psychopathic personality traits in normal population (e.g., Sellbom et al., 2018; Sleep et al., 2019); (3) the relationship between the constructs that make up the nomological network of psychopathy (e.g., violence, criminality, antisocial behavior, alcohol abuse, etc.) and the five-factor model or instruments that measure psychopathic personality traits in normal population (e.g., Watt and Brooks, 2012; Vize et al., 2018; Sleep et al., 2019); and (4) studies that have examined through taxonomic methodology whether psychopathy is a dimensional or a categorical construct (e.g., Guay et al., 2007, 2018).

Reviewing all these lines of research is beyond the scope of this work, but, in general, the data of all of them seem to support a dimensional view of psychopathy, which conceives it as a maladaptive variant of the normal personality. As a result, in recent years, there is strong interest in studying the presence and influence of psychopathy in everyday life, from the working world to couple relationships (Hare, 2003b; Dutton, 2012; Babiak and Hare, 2019; Fritzon et al., 2020). The scientific literature acknowledges the existence of people with high levels of psychopathy who are not offenders or violent, the so-called “integrated psychopaths.” It also acknowledges the existence of people with high levels of psychopathy who achieve great success in their lives, the so-called “successful psychopaths.” The construct of “successful psychopathy” refers to those psychopathic personality traits such as lack of fear, high self-confidence or charisma, which can be beneficial in certain contexts (Dutton, 2012; Lilienfeld et al., 2015).

In this direction, it has been proposed that it would be possible to find higher levels of psychopathic traits in certain professions or occupations (e.g., entrepreneurs, managers, politicians, investors, salesmen, surgeons, lawyers, telemarketing employees). The reason behind this could be that it is precisely these traits the ones that could boost the tasks involved in those professions or occupations and even facilitate success in them (Hare, 2003b; Dutton, 2012; Babiak and Hare, 2019; Fritzon et al., 2020). Among these professions, some are typically related to office workers, the so-called “white-collar workers;” hence, the term “white-collar psychopathy” has been coined, although the term “corporate psychopathy” or “organizational psychopathy” is also used.

In this regard, Dutton (2012), after applying the Levenson Self-Report Psychopathy Scale (LSRP; Levenson et al., 1995) online to 5,400 people and asking about their profession, found that, in the United Kingdom, the 10 professions with the highest levels of psychopathic traits were company CEOs, lawyers, radio or television characters, salespersons, surgeons, journalists, priests, police officers, chefs, and civil servants. On the other hand, the 10 professions with the lowest levels of psychopathic traits were social-health assistants, nurses, therapists, artisans, stylists, charity workers, teachers, creative artists, physicians, and accountants. In the same direction, Lilienfeld et al. (2014) applied the brief version of the Psychopathic Personality Inventory-Revised (PPI-R-SF; Lilienfeld and Widows, 2005), obtaining valid responses from 3,388 people. What they found was that people in a managerial position at work had higher levels of psychopathic traits than those who did not occupy such positions, and that those who worked in jobs with occupational risk (e.g., police officers, firefighters, military service people, miners) also had higher levels of psychopathic traits than those working in jobs without occupational risk. Employers also showed higher levels of psychopathic traits compared to psychologists and other mental health professionals.

In this theoretical and empirical context, the main objective of this work was to systematically review the scientific literature on the prevalence of psychopathy in the general population. By doing so, we have obtained an estimate of its prevalence rate in that population because, to our knowledge, no studies have reviewed that scientific literature to date. In this sense, this work hypothesizes that this prevalence will be much lower than the one observed in the offender or prison population. Secondarily, the present work was intended to examine the influence of the following factors on the prevalence of psychopathy in the general population: the type of instrument used to evaluate and define psychopathy, people's sex, their profession-occupation, and their country of origin. In this line, and based on the scientific literature presented above, this work hypothesizes that the prevalence rates will be lower using the PCL-R (or any of its versions) than with other instruments; they will be higher in males than in females; higher in samples of employees and managers of certain commercial or financial organizations or companies than in other types of samples obtained from the general population; and higher among people from North American countries than in people from European countries.

The present review was focused on the prevalence of psychopathy as defined by the authors of the reviewed studies, but it did not examine the prevalence of psychopathic personality traits. Therefore, and as it will be explained in more detail later, studies about the prevalence of psychopathic personality traits or psychopathic facets (e.g., meanness, disinhibition, fearless dominance, psychopathic interpersonal facet) that did not define the presence of psychopathy based on those traits or facets were excluded from the review.

## Methods

### Identification of Publications

To find studies relevant to the objectives of this work, on June 20, 2021, a bibliographic search was carried out in PsycInfo, MEDLINE, and PSICODOC. These are currently the most complete bibliographic databases in psychology, medicine, and psychology in Spanish, respectively. The search was performed in PsycInfo and MEDLINE using the following words in any field of the databases: *community* or *university* or *college* or *normal* or *non-criminal*, combined with these three terms in the summary or document title fields: *prevalence* and *psychopathy* or *psychopathic trait*. When conducting the search in PsycInfo and MEDLINE, the following expression was specifically used: *(ab((psychopathy or “psychopathic trait”) and prevalence) or ti((psychopathy or “psychopathic trait”) and prevalence)) and (community or university or college or normal or non-criminal)*. The search in PSICODOC was performed by combining the following pairs of words (in Spanish) in any field of the database: *prevalence* and *psychopathy* or *prevalence* and *psychopath* or *prevalence* and *psychopathic*. In particular, the following expression was used in the PSICODOC search: *(prevalence and psychopathy) or (prevalence and psychopath) or (prevalence and psychopathic)*.

**Figure 1:**
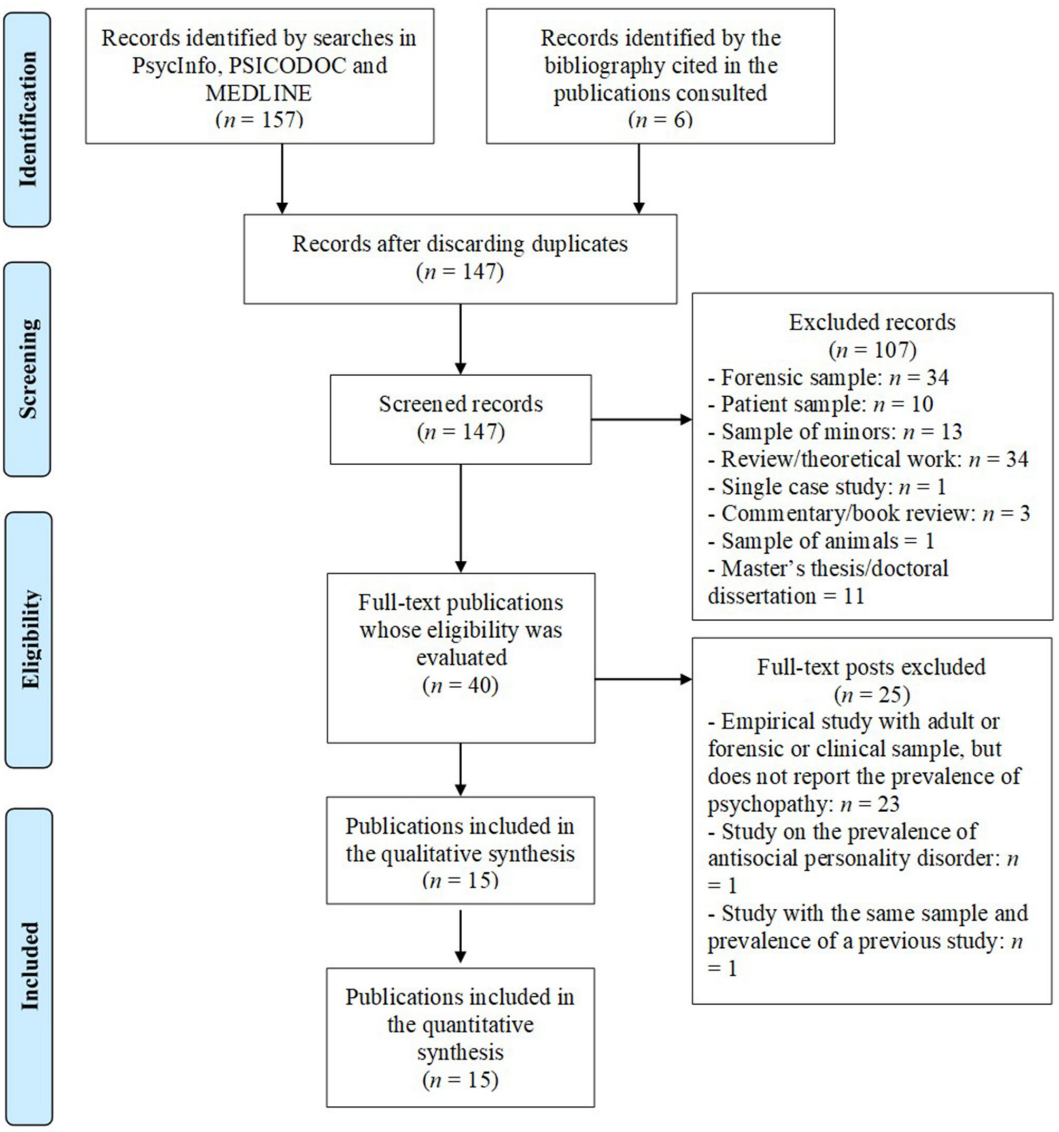
Flowchart of the process of searching and selecting studies on the prevalence of psychopathy in the general adult population.

Previous searches identified 157 publications. After discarding duplicates that appeared in two or more bibliographic databases and adding six different publications identified after consulting the literature cited in the consulted studies, 147 publications were obtained. Figure 1 presents a summary using the flowchart proposed by the PRISMA group for the publication of systematic reviews of the scientific literature (Moher et al., 2009). It also shows the process followed for the search, screening, and selection of studies on the prevalence of psychopathy in the general adult population.

### Screening of Publications

Based on the objectives of this work and the reading of the titles, basic bibliographic data, and abstracts, the 147 publications initially found were screened to determine whether they met the following inclusion criteria: (1) it reports an empirical study that provides data on the prevalence of psychopathy; (2) it reports a study carried out with samples of adult participants from the general population, including community samples, organizations or companies, university students, private professions, etc., but not clinical samples or samples from forensic or prison contexts; (3) it describes the procedure used to define and evaluate psychopathy; and (4) it is a journal article, book, or book chapter. Doctoral dissertations, master's theses, and technical reports were excluded because of the difficulties sometimes encountered to achieve their full text. Presentations at congresses were also excluded because of the limited information on the studies they usually present.

Therefore, as shown in Figure 1, publications reporting theoretical studies or literature reviews (34 publications) were excluded. Three publications that reviewed other works (book reviews) or submitted commentaries on other studies were also excluded. Publications reporting single-case studies (one publication) or reporting studies of samples of participants from the forensic population (34 publications), samples of patients with psychological or physical disorders (10 publications), samples of children or adolescents (13 publications), or animal samples (one publication) were also excluded. Finally, 11 publications that were doctoral dissertations or master's theses were excluded. Following these exclusions, the sample of publications was reduced to 40.

### Study Eligibility

Of the 40 publications resulting from the screening phase, their full text was obtained, and, based on their reading, these publications were re-evaluated for compliance with the inclusion criteria. As reflected in Figure 1, 23 publications were excluded at this stage because, although they reported empirical studies of samples of adult participants from the general population, they did not provide data on the prevalence of psychopathy in these samples. Some of these publications reported empirical studies on the prevalence of some psychopathic personality traits or psychopathic facets, but, since they did not define the presence of psychopathy based on those traits or facets, they were excluded from the review. For example, Neumann et al. (2012) calculated the proportion of adults from the general population across sex and world region that showed a high level for each facet of the Self-Report Psychopathy Scale (SRP) of Hare: interpersonal, affective, lifestyle, and antisocial. However, Neumann et al. (2012) did not define psychopathy based on SRP facets. For example, they did not establish if it is necessary to show high levels in all four SRP facets to identify psychopathy, or if it is necessary to show high levels in only three, two or one of the four facets. It is also not indicated if it is necessary to show a high level in a particular facet—e.g., antisocial—in addition to high level in one or two of the remaining facets. Therefore, Neumann et al. (2012) did not report data on the prevalence of psychopathy and their study was excluded from the present review.

Finally, two more publications were excluded. One of these publications offered data on the prevalence of antisocial personality disorder, but not the prevalence of psychopathy. The second publication reported a study conducted with the same sample of adults from the general population who participated in a previously published study. Given that it offered the same psychopathy prevalence data as that prior study, it was also excluded.

### Studies Included in the Review

Following the screening and eligibility process of the initially identified publications, this systematic review finally included 15 publications reporting 15 studies. The main characteristics of these studies in terms of their participants and the definition of psychopathy used are summarized in Table 1.

**Table 1:** Main characteristics of studies on the prevalence of psychopathy in the general adult population analyzed in this systematic review.

**References**	**Participants**	**Definition of psychopathy**
Babiak et al. (2010)	203 managers and executives from the USA (77.8% male; mean age = 45.8 years)	Total score of ≥ 30 in the PCL-R (subclinical psychopathy: ≥ 25)
Boduszek et al. (2021)	1,201 adults from the general population (32% male; mean age = 32.2 years) and 2,080 university students (26.3% male; mean age = 20.3 years) from the UK	Identification, through latent profile analysis with the four dimensions of the PPTS, of a group with high scores (“high psychopathy group”)
Coid et al. (2009)	638 residents in private homes in the UK, aged between 16 and 74 (43.3% male; range/mean age = 16–74/45.4 years)	Total score of ≥ 18 in the PCL:SV (subclinical psychopathy: ≥ 13)
Fritzon et al. (2017)	261 purchasing and supply professionals, members of an Australian organization (61.7% male; range/mean age = 27–75/47.9 years)	Total scores ≥ 65 T in the PPI-R
Gordts et al. (2017)	1,510 adults in the general population of Belgium (48% male; range/mean age = 17–90/33.7 years)	Total score > 70 T in the SRP-III
Gustafson and Ritzer (1995)	214 university students (40% male) and 367 university students (30% male) from the USA.	Identification, through cluster analysis with the total scores of five instruments of psychopathic traits, of a group that fits the psychopathic profile
Hagnell et al. (1994)	2,036 adults from a Swedish town, between 20 and 80 years of age evaluated in 1972 (49.9% male)	Diagnosis by a psychiatrist based on a clinical interview, with a free part and a structured part, without reliability or validity data
Levenson et al. (1995)	487 university students from the USA (28% male; mean age = 20.8 years)	Scores of ≥ 3 on at least 8 of the 16 primary psychopathy items of the LSRP
Love and Holder (2014)	427 Canadian university students (31.1% male; mean age = 20.2 years)	Scores of ≥ 3 on at least 8 of the 16 primary psychopathy items of the LSRP
Neumann and Hare (2008)	514 adults from the USA (38.1% male; range/mean age = 18–40/31 years)	Total score of ≥ 13 on the PCL:SV (psychopathy: ≥ 18)
Pethman and Erlandsson (2002)	292 Swedish university students (22% male; mean age = 27.5 years)	Identification, through cluster analysis with the total scores of five instruments of psychopathic traits, of a group that fits the psychopathic profile
Robitaille et al. (2017)	224 adult males from the general population of Canada evaluated at age 33 and without borderline or antisocial personality disorders	Total scores of ≥ 30 on the PCL-R
Salekin et al. (2001)	326 university students from the USA (44% male; mean age = 22 years)	A proportion of positive responses on the SRP-II or the PPI that correspond to the proportion needed for the diagnosis of psychopathy on the PCL-R
Seara-Cardoso et al. (2020)	513 students and workers from several universities in Portugal (26% male; range/mean age = 16–60/27.4 years)	Total score > 70 T on the SRP-SF
Spencer and Byrne (2016)	204 workers from an Australian advertising agency (51.5% male; age range = 18–69 years; 9.8% senior managers, 42.1% mid-level managers, and 48% low-level employees)	Scores of > 70 T on the LSRP

*Note: LSRP, Levenson Self-Report Psychopathy Scale (Levenson et al., 1995); PCL-R, Hare Psychopathy Checklist-Revised (Hare, 1991, 2003a); PCL:SV, Hare Psychopathy Checklist-Screening Version (Hart et al., 1995); PPI, Psychopathic Personality Inventory (Lilienfeld and Andrews, 1996); PPI-R, Psychopathic Personality Inventory-Revised (Lilienfeld and Widows, 2005); PPTS, Psychopathic Personality Traits Scale (Boduszek et al., 2016); SRP-II, Self-Report Psychopathy Scale-II (Hare, 1990); SRP-III, Self-Report Psychopathy Scale-III (Paulhus et al., 2016)*.

### Statistical Analysis

The meta-analytical calculations to estimate the joint prevalence of psychopathy and the heterogeneity and bias indices of the results were performed using MetaXLversion 5.3 (EpiGear International, Sunrise Beach, QLD, Australia), whereas the meta-regression analyses were performed with Stata, version 15 (Stata Corp, College Station, TX, USA) based on the data provided by MetaXL.

To stabilize the variances, the double arcsine transformation of the prevalences was used to calculate the conjoint prevalence, and an inverse variance heterogeneity model was used, as this model uses robust error variances (Barendregt et al., 2013; Wang and Liu, 2016). A random-effects model was also used but, as its results were virtually the same, they are not presented in this article for the sake of brevity. To facilitate the interpretation of the results, individual and joint prevalences are presented in the graphs as proportions after reversing the applied transformations. To assess the heterogeneity between the studies, Cochran's *Q* test and the *I*^2^ statistic were calculated, and to evaluate the publication biases of the meta-analysis, we used the Doi chart and the Luis Furuya-Kanamori index (LFK), which has been shown to be better than the plot funnel and Egger's linear regression test for skewness detection and, hence, publication biases (Furuya-Kanamori et al., 2018).

The combined prevalences for different subgroups of studies created based on the following moderator variables were also calculated, following the above methods: type of instrument used to measure psychopathy (PCL-R vs. other instruments), participants' sex (male vs. female), type of samples of the general population (organizations vs. community vs. university students), and country of origin of the samples (North America vs. United Kingdom-Australia vs. mainland Europe). The significance of the differences in the joint prevalences of the different subgroups was tested with individual meta-regression analyses for each of these moderator variables and through a multiple meta-regression analysis with all the moderator variables that were statistically significant in individual analyses. In the case of the two moderator variables with three categories (sample type and country), two binary dummy variables were created for each of them for the meta-regression analyses.

## Results

### Characteristics of the Studies

A total of 16 unique samples of participants from 15 studies were used to estimate the prevalence of psychopathy in the general adult population because, in one of the studies listed in Table 1 (Boduszek et al., 2021), a sample of adults from the population and another sample of university students participated, obtaining differentiated psychopathy prevalence rates for each of these two samples.

The 16 samples included a total of 11,497 people who were mostly university students, with seven samples (43.75% of the total samples), adults of the community, with six samples (37.5%), and the remaining three samples of participants recruited from different organizations (18.75%).

The samples of participants came mainly from the USA, with five samples (31.25%), the United Kingdom, with 3 samples (18.75%), Canada, with two samples (12.5%), Australia, with two samples (12.5%), and Sweden, with two samples (12.5%), with the remaining two samples from Portugal and Belgium. To study the influence of the country of origin on the prevalence of psychopathy, the samples were grouped into three categories: North America (USA and Canada: 43.75%), the United Kingdom-Australia (31.25%), and mainland Europe (Sweden, Belgium, and Portugal: 25%).

In nine of the 15 studies listed in Table 1, psychopathy was evaluated and defined with self-reporting instruments (60% of the studies), whereas in the five other studies, it was done with clinician rating instruments (33.3%). In the remaining study it was done through an interview (6.7%). Among the self-reporting instruments, the most commonly used were the LSRP (Levenson et al., 1995) and the SRP of Hare in its different versions (SRP-II, SRP-III, and SRP-SF; Hare, 1990; Paulhus et al., 2016), each used in three studies, meaning it was used in 66.6% of the self-report studies. With regard to the clinician rating tools, all studies using these instruments used the PCL-R or one of its versions (specifically, the PCL:SV).

Finally, most of the 15 reviewed studies were published in the twenty-first century (12 studies: 80%), especially in the last 10 years (2010–2021: 8 studies, 53.3%), with the three oldest studies published in 1994 and 1995.

### Prevalence of Psychopathy in the General Population

**Figure 2:**
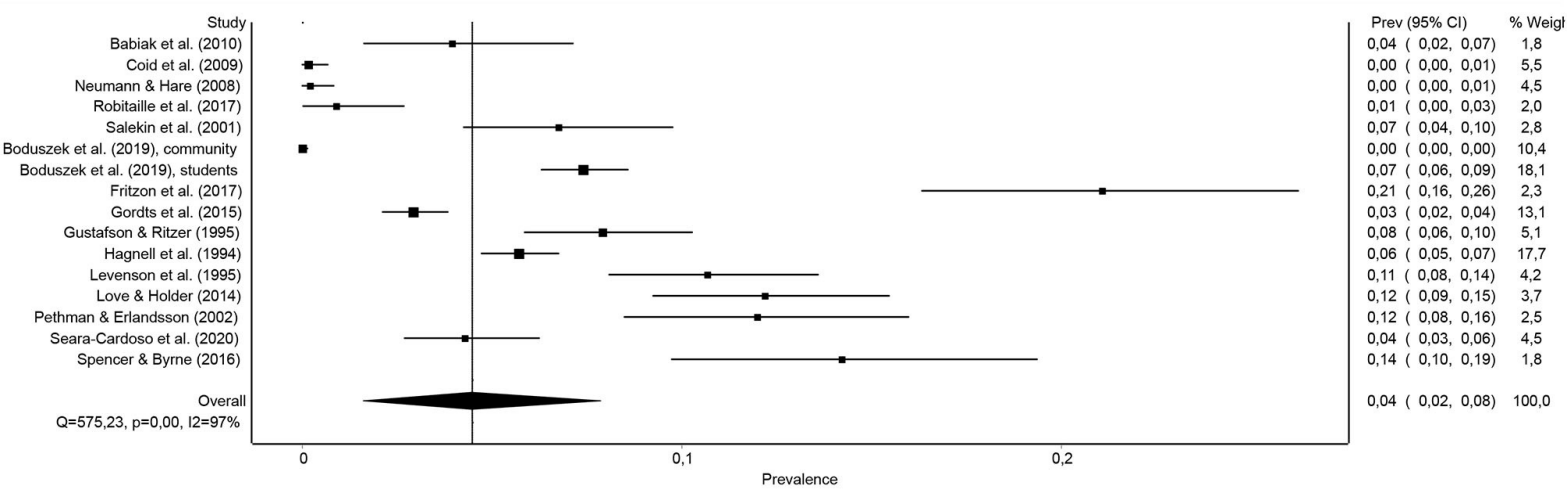
Forest plot of the prevalence (in proportions) of psychopathy in the general population.

Figure 2 presents the prevalence rates of psychopathy found in the 16 samples of the general population analyzed in the present work, and also the joint prevalence rate. The sample prevalence rates ranged from 0 to 21%, with the combined prevalence of 4.5% [95% CI (1.6, 7.9%)].

**Figure 3:**
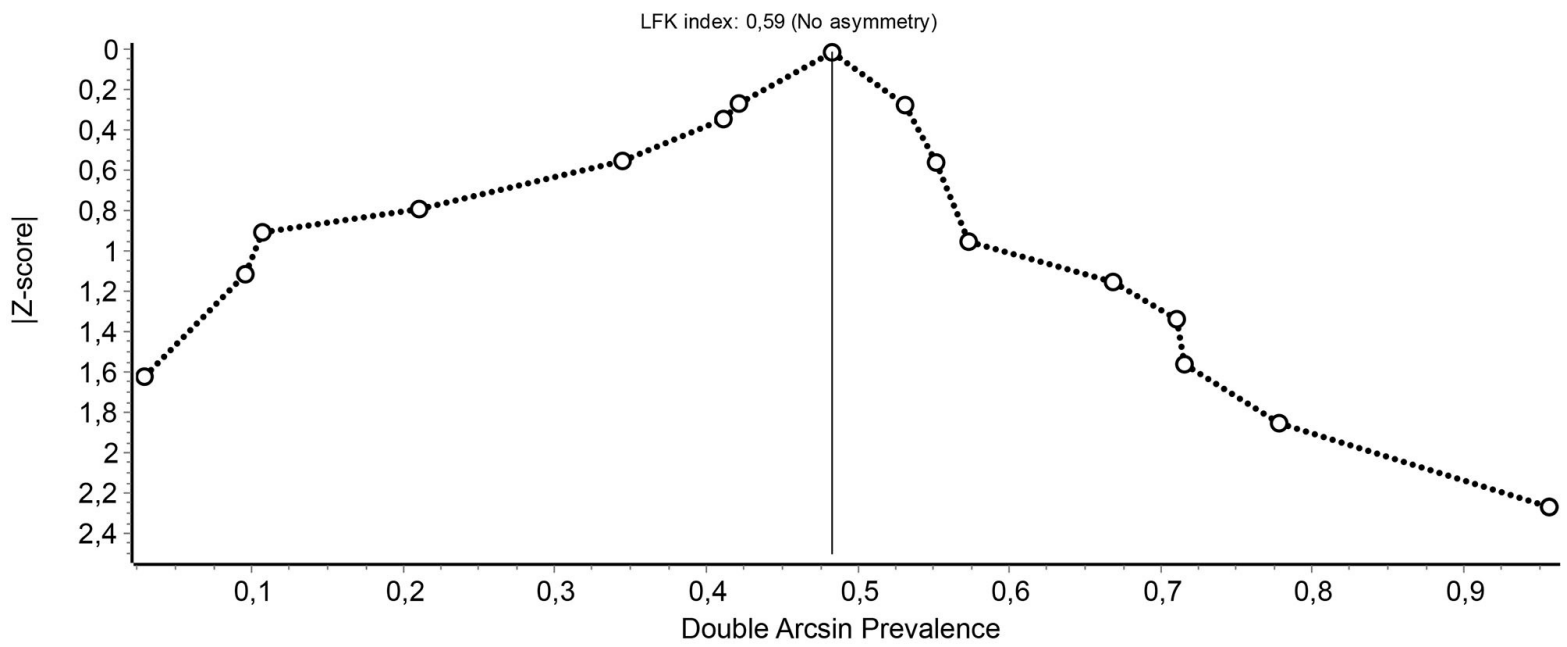
Indicators to evaluate the skewness of the results of the studies and detect the presence of publication biases: Doi chart and LFK index obtained in studies on the prevalence of psychopathy in the general population.

The heterogeneity between the samples was noticeable, as the statistic *I*^2^ = 97.4% [95% CI (96.6, 97.9%)] and a statistically significant *Q-*test (*Q* = 575.22, *p* < 0.001) were obtained, which justified the analysis of the moderator variables that could partly explain such heterogeneity. Moreover, both the Doi chart and the LFK index suggested that this heterogeneity did not seem to translate into a large asymmetry that could reflect a significant publication bias, because, for example, the LFK index obtained (0.59) was in the range of 1/−1, indicating no asymmetry (see Figure 3).

### Prevalence of Psychopathy Based on Moderator Variables

The results of the individual meta-regression analyses performed on the prevalence of the 16 samples of the general population indicated that the type of instrument [*F*_(1, 14)_ = 5.24, *p* < 0.038, *R*^2^ = 0.17] and the type of sample [*F*_(2, 13)_ = 4.96, *p* < 0.025, *R*^2^ = 0.52] significantly influenced the prevalence of psychopathy, but not the country of origin of the samples [*F*_(2, 13)_ = 0.16, *p* = 0.857, *R*^2^ = 0.03].

**Figure 4:**
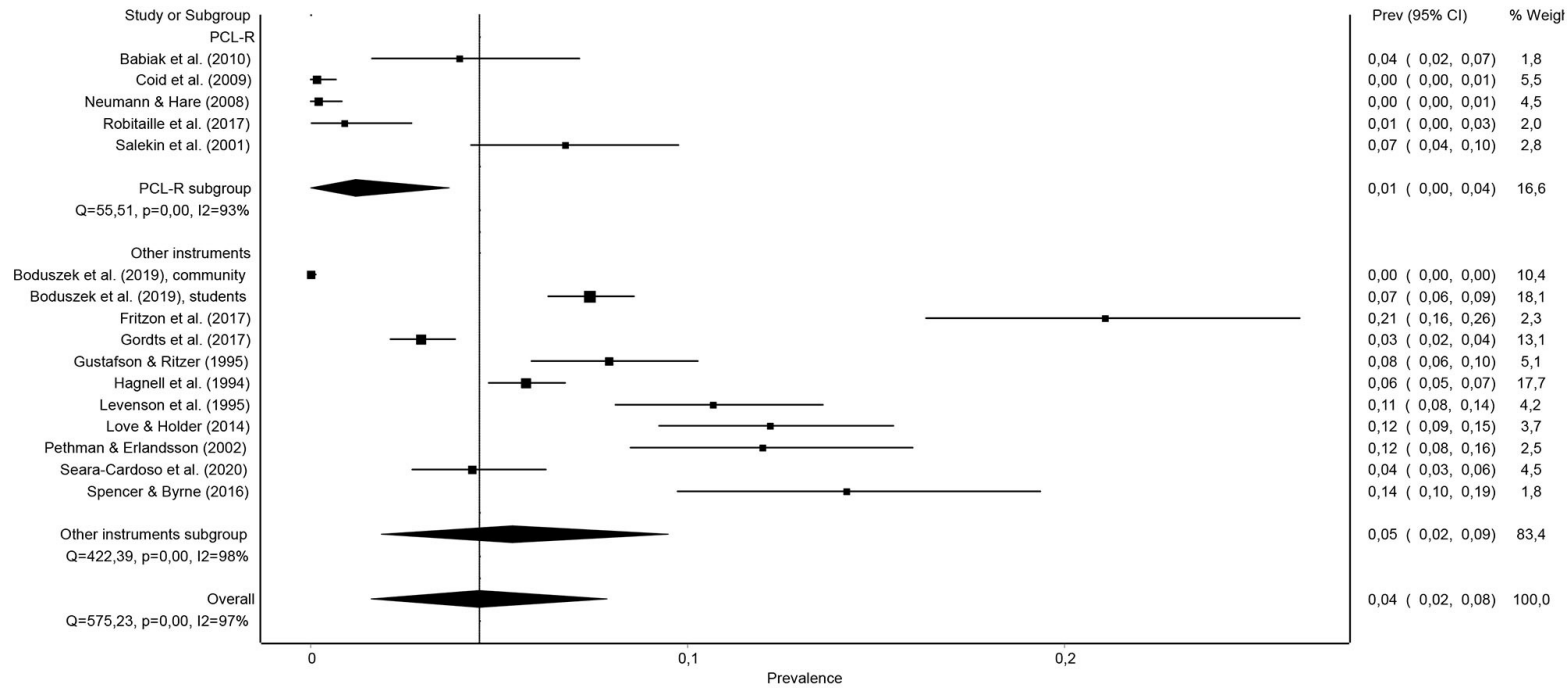
Forest plot of the prevalence (in proportions) of psychopathy in the general population depending on the type of instrument used to measure psychopathy: PCL-R vs. other instruments.

As can be seen in Figure 4, studies using instruments other than the PCL-R found much higher rates of psychopathy prevalence (more than triple or quadruple, on average) than studies that used the PCL-R or any of its versions, with the combined prevalence of 5.4% [95% CI (1.9, 9.5%)] in the former case, and only of 1.2% [95% CI (0–3.7%)] when using the PCL-R or any of its versions.

**Figure 5:**
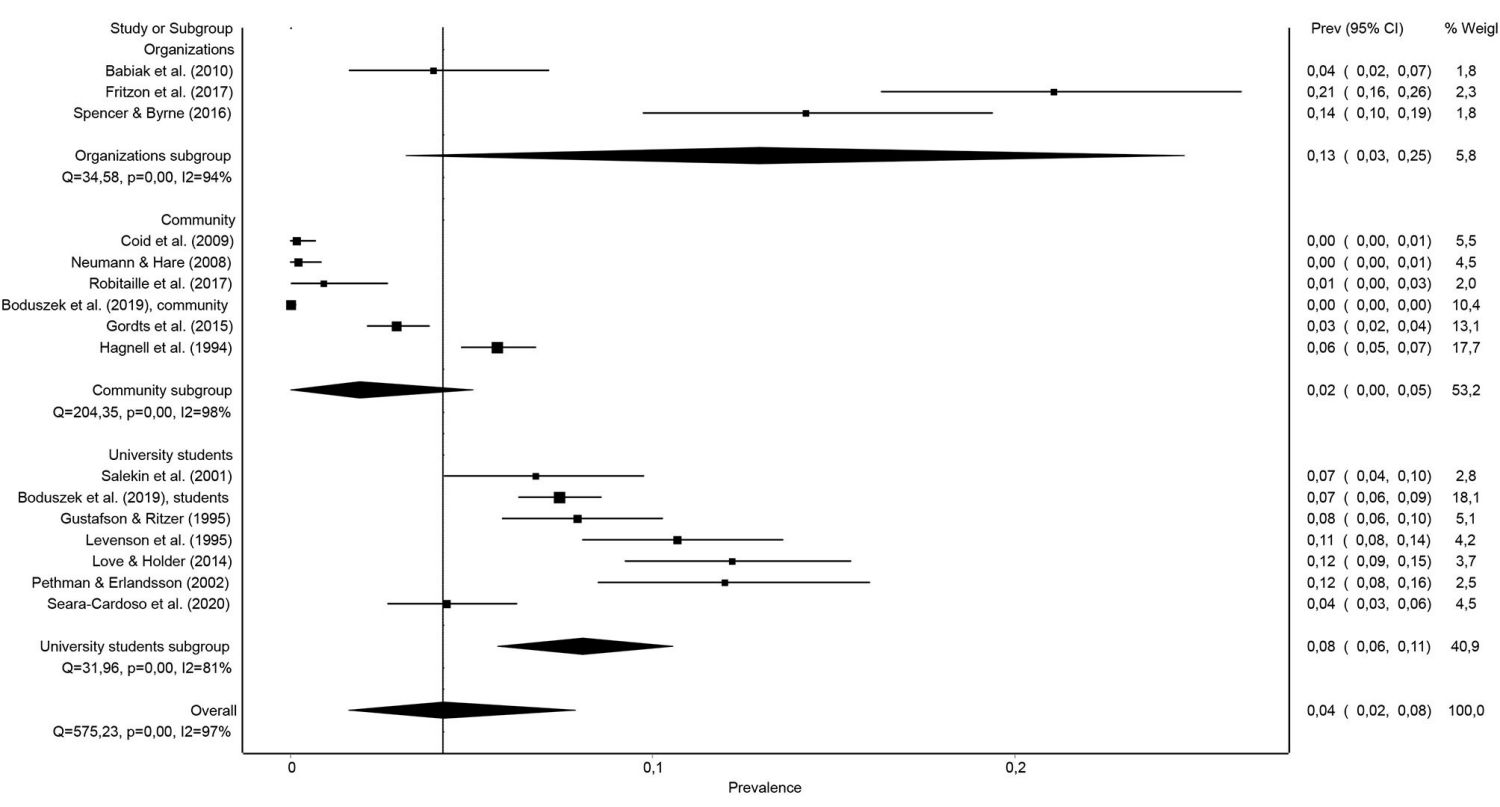
Forest plot of the prevalence (in proportions) of psychopathy in the general population depending on the type of sample evaluated: organizations, community, or university students.

For its part, the combined prevalence of psychopathy in samples of organizations [12.9%, 95% CI (3.2, 24.7%)] was higher than in samples of university students [8.1%, 95% CI (5.7, 10.6%)] or in community samples [1.9%, 95% CI (0, 5%)] and, in turn, the joint prevalence among university students was generally higher than among people in the community (8.1 vs. 1.9%) (see Figure 5). In fact, in the individual meta-regression analysis, the two binary dummy variables created were statistically significant: organizations vs. non-organizations (β = 0.458, *p* < 0.022) and college students vs. non-college students (β = 0.297, *p* < 0.013).

Moreover, when both the type of instruments and the type of samples were included in a multiple meta-regression analysis that, predictably, yielded a statistically significant model [*F*_(3, 12)_ = 32.09, *p* < 0.0001, *R*^2^ = 0.63], only the two binary variables related to the sample type (organizations vs. non-organizations and university students vs. non-university students) continued to be significantly related to the prevalence of psychopathy (β = 0.474, *p* < 0.003 and β = 0.266, *p* < 0.032, respectively), whereas the type of instrument did not reach statistical significance (β = 0.198, *p* < 0.055).

As only 5 of the 15 studies listed in Table 1 offered separate prevalence rates for male and female subsamples (Hagnell et al., 1994; Levenson et al., 1995; Salekin et al., 2001; Neumann and Hare, 2008; Coid et al., 2009; Love and Holder, 2014) and in a sixth study, the sample was entirely male (Robitaille et al., 2017), the role of sex as a moderator could only be examined in those six studies, which provided, in total, 13 subsamples/samples.

**Figure 6:**
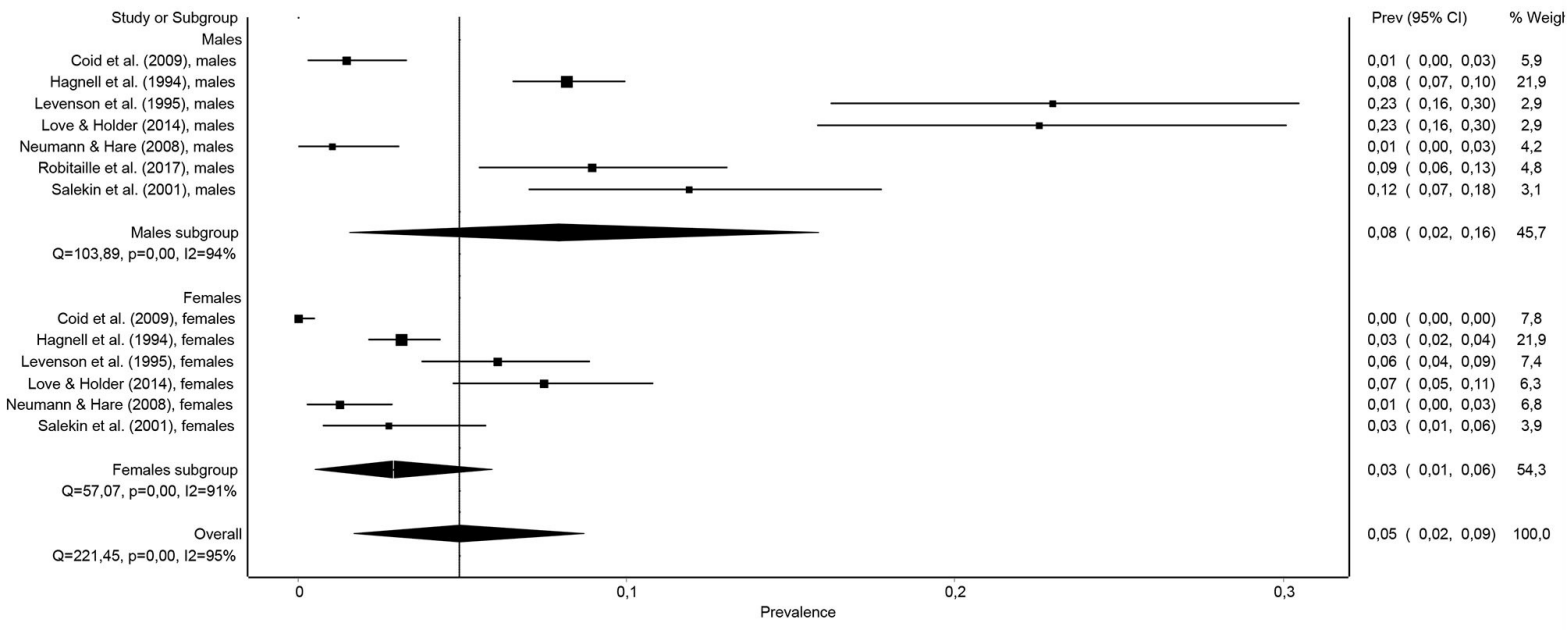
Forest plot of the prevalence (in proportions) of psychopathy in the general population based on gender.

The results of the individual meta-regression analysis on the prevalence of these 13 subsamples/samples revealed that sex significantly influenced the prevalence of psychopathy, *F*_(1, 11)_ = 6.00, *p* < 0.032, *R*^2^ = 0.27. In particular, the combined prevalence of psychopathy was higher (more than double) in males [7.9%, 95% CI (1.6, 15.8%)] than in females [2.9%, 95% CI (0.5, 5.9%)] (see Figure 6).

In fact, in the above 13 subsamples/samples, sex was the only moderator significantly related to the prevalence of psychopathy, as neither the country of origin [North Americans vs. Europeans, including the United Kingdom: *F*_(1, 11)_ = 1.08, *p* = 0.320, *R*^2^ = 0.08], nor the type of instrument [*F*_(1, 11)_ = 4.17, *p* = 0.065, *R*^2^ = 0.25], nor the type of sample [university students vs. community: *F*_(1, 11)_ = 4.22, *p* = 0.064, *R*^2^ = 0.24] proved to be significant in their respective individual meta-regression analyses. However, it should be noted that, in the case of the last two variables, the results showed a trend toward statistical significance that may have been impaired by the fewer subsamples/samples compared with the overall analysis with all the global samples of Table 1 (13 vs. 16 samples).

## Discussion

The main objective of this study was to obtain an estimate of the prevalence of psychopathy in the general adult population and, in this sense, to our knowledge, it is the first systematic or meta-analytic review carried out on this topic. Following a thorough search in the scientific literature, 15 empirical studies were found that had calculated the frequency of psychopathy in samples from the general adult population, including community, organization, and university student samples. These studies used properly described tools and procedures to assess and define psychopathy. After calculating the conjoint mean of their results with meta-analytic procedures, based on a total sample of 11,497 people, it can be estimated that the prevalence of psychopathy in the general adult population is 4.5%.

As could be expected, this prevalence is much lower than that found in samples obtained in forensic or prison contexts. For example, in the meta-analysis of Fox and DeLisi (2019), it was found that the average prevalence of psychopathy among homicide offenders could be estimated at 27.8 or 34.4%, depending on the criterion used to define psychopathy with the PCL-R (cut-off score of 30 vs. 25, respectively). In the second edition of the PCL-R manual (Hare, 2003a), the prevalence of psychopathy, based on a cut-off score of 30, was 15.7% for males (Nicholls et al., 2005) and 10.3% for females (Guay et al., 2018) in the North American normative samples of prisoners.

However, although the average prevalence of psychopathy in the general population is clearly lower than that found in the offender or prison population, the prevalence rates of psychopathy in the general population obtained in the studies reviewed in this work show considerable variation, ranging from a minimum of 0% to a maximum of 21%. In fact, the results obtained in terms of the *I*^2^ and *Q* statistics confirmed that the heterogeneity of the studies was statistically significant.

These variations depend on many factors, such as the role of the type of instrument used to define psychopathy, the participants' sex, the type of sample of the general population, and the participants' country of origin. These factors have been analyzed in this work. In this sense, the results of the present work indicate that the first three factors, but not the country of origin, seem to have a significant impact on the prevalence of psychopathy. Depending on the chosen instrument, the participants' sex or the type of sample selected, prevalence figures can double, triple, or quadruple the figures found with a different instrument or with participants of another sex or from a different subpopulation of the general population. Moreover, the results obtained in terms of the Doi chart and the LFK index indicate that this heterogeneity does not appear to reflect a significant publication bias, but could largely be attributed to these three moderator variables.

In particular, the results of this work indicate that, when using the PCL-R (or any of its versions), an instrument that is currently considered as the gold standard for the evaluation and definition of psychopathy, it can be estimated that the prevalence of psychopathy in the general adult population is only 1.2%. However, if other instruments are used, such as self-reports of psychopathic personality traits like the LSRP (Levenson et al., 1995) or the SRP in their different versions (SRP-II, SRP-III, and SRP-SF; Hare, 1990; Paulhus et al., 2016), the estimate of the prevalence of psychopathy in the general adult population quadruples, reaching 5.4%.

In fact, as virtually all the studies with offenders use the PCL-R or one of its versions, the comparison between the prevalence rates of psychopathy obtained in the general population and in the offender or prison population should primarily focus on studies conducted with the PCL-R. In this sense, the difference in the prevalence rate of psychopathy between the two types of population, general and criminal, is much greater: 1.2%, obtained in the present work for general population, compared to 15.7 and 10.3%, obtained in the normative samples of the PCL-R for male and female prisoners, respectively (Nicholls et al., 2005; Guay et al., 2018), or vs. 27.8%, obtained in Fox and DeLisi (2019) meta-analysis for homicide offenders.

Differences in the prevalence rates as a function of the type of instrument and cut-off point established to identify psychopathy go back to the problems in defining the construct of psychopathy. Those differences also point out a limitation of the present study. We will elaborate on these ideas later in the context of the limitations of this review.

The results of this study also indicate that the prevalence of psychopathy in the general adult population is significantly higher among males than among females. In particular, psychopathy in the general population doubles its prevalence in males compared to females (7.9 vs. 2.9%). This difference is consistent with the results obtained in samples of offenders or incarcerated people, among whom the prevalence of psychopathy is also higher in males than in females (Beryl et al., 2014).

In particular, Beryl et al. (2014) conducted a systematic review of the scientific literature on the prevalence of psychopathy in adult women from within secure settings, which included criminal justice settings, or secure inpatient healthcare settings. They found prevalence rates ranging from 0 to 31% using the PCL-R or one of its versions, although they did not report the average of these rates or the conjoint prevalence. However, from the data they submitted for females in criminal justice settings, it is possible to calculate, for the 13 unique studies that defined psychopathy based on a cut-off score of 30 in the PCL-R or of 18 in the PCL:SV, a weighted average prevalence of 11.9% (Table 3 of Beryl et al., 2014, p. 191). This figure dropped slightly to 11% when also taking into account the data from the 10 unique studies that had evaluated samples of females in secure/inpatient psychiatric settings or mixed samples—secure/inpatient psychiatric and criminal justice settings—(Tables 2, 4, respectively, of Beryl et al., 2014, p. 190, 192). Moreover, these figures hardly varied when only studies using the same instrument, the PCL-R, and the same cut-off score, 30 (12.3 and 11.4%, respectively) were taken into account. Interestingly, these prevalence figures are very similar to those presented by the scales of female prisoners collected in the second edition of the PCL-R manual, which, as noted above, show a prevalence of psychopathy in female prisoners of 10.3% (Guay et al., 2018). In summary, the average prevalence of psychopathy in female offenders or prisoners can be estimated at 10–12%.

In contrast, in male offenders or prisoners, using the PCL or its versions, rates of average prevalence of psychopathy of 15–35% are usually obtained, although the average rates of 15–25% are probably the most adequate (Hare, 1991, 2003a; Guay et al., 2007; Fox and DeLisi, 2019, cited by Nicholls et al., 2005). In the 1991 PCL-R manual, Hare reported that, in a global sample of 1,200 males incarcerated in Canadian prisons, 25% scored 30 or higher on the PCL-R. However, in the second edition of the PCL-R manual, published in 2003 and based on a much larger sample with a total of 5,408 males incarcerated in American prisons, Hare reported that 15.7% of the inmates scored 30 or higher on the PCL-R (Hare, 1991, 2003a; cited by Nicholls et al., 2005). Subsequently, with that same large sample, but eliminating the participants with missing information on some items of the PCL-R (*n* = 543), Guay et al. (2007) reported that 19% of the remaining 4,865 male inmates scored 30 or higher on the PCL-R. Finally, in the meta-analysis of Fox and DeLisi (2019), it was found that 27.8% of the homicide offenders scored 30 or higher on the PCL-R.

In any case, it seems clear that the prevalence of psychopathy is higher in male offenders or prisoners than in female offenders or prisoners (15–25% vs. 10–12%), and this difference between the sexes is maintained in the general population (7.9 vs. 2.9%), as shown in this meta-analysis.

Another interesting result of this work has to do with the finding of differences in the prevalence of psychopathy between different groups of adults in the general population. In particular, this review has found that the prevalence of psychopathy is significantly higher among workers in some organizations and companies (managers, executives, procurement and supply professionals, advertising workers) than among university students or among people from the general community (12.9 vs. 8.1% and 1.9%, respectively). In turn, the prevalence among university students is significantly higher than among people from the general community (8.1 vs. 1.9%).

The highest prevalence of psychopathy among workers in certain organizations and companies is based on data from only three studies with a total sample of 668 people and should, therefore, be taken with some caution. However, this result is consistent with the scientific literature that proposes that psychopathy is more prevalent in certain professions (e.g., entrepreneurs, managers, politicians, investors, sellers, surgeons, lawyers, telemarketing employees) in which the personality characteristics that define psychopathy could even facilitate their success in these professions (Hare, 2003b; Dutton, 2012; Babiak and Hare, 2019; Fritzon et al., 2020).

More surprising may be the result that among university students, there is a higher prevalence of psychopathy than among people in the community. Following the previous argument, it could be assumed that among university students of certain professions there could be more people with psychopathic traits (e.g., students in business administration and management, marketing), but it could also be assumed that among university students from other professions, there could be more people with less psychopathic traits and characterized, on the contrary, by high levels of empathy, altruism, candor, trust, humility, and responsibility (e.g., students from health professions, social work, and other professions closely linked to helping). In fact, in a study of Hassall et al. (2015), it was found that business university students, in comparison to university students of psychology, showed significantly higher levels in the four psychopathy factors measured by the SRP-III (Paulhus et al., 2016). Unfortunately, this work did not provide data on the prevalence of psychopathy in the two groups of university students. In addition, in the study of Dutton (2012), mentioned in the Introduction, among the 10 professions with higher levels of psychopathic traits, there were some that require a university degree (e.g., lawyer, surgeon, journalist) and, likewise, among the 10 professions with lower levels of psychopathic traits, there were also several that require a university degree (e.g., nurse, teacher, doctor).

Therefore, future research with university students should examine whether there are significant differences in psychopathy among students of different careers. This implies that, not only among university students of certain careers may there be a higher prevalence of psychopathy than in the general population, but that among university students of other careers, there may be a similar prevalence. It could even be that among university students of certain careers, there may be a lower prevalence of psychopathy than in the general population.

Research on differences in psychopathy between people of different professions or between university students of different careers departs from the traditional application of the construct of psychopathy to the forensic and prison area. That research intertwines, as discussed in the Introduction, with the most recent interest in the presence of psychopathy in everyday life (Dutton, 2012; Babiak and Hare, 2019; Fritzon et al., 2020), in the definition of psychopathy in terms of normal personality models such as the Big Five model (Lynam and Miller, 2019), and in the concept of successful or integrated psychopathy (Dutton, 2012; Lilienfeld et al., 2015). The fact that, as found in this review, most studies on the prevalence of psychopathy in the general population were published in the twenty-first century, especially in the last 10 years, is also consistent with those most recent interests far from the area of forensic and prison psychology.

Finally, no significant differences in the prevalence of psychopathy in the general population were found in this work as a function of the country of origin of the evaluated people. This absence of differences is not consistent with the results of the scientific literature on criminal and prison populations, which show the existence of differences between countries, especially between North American and European countries, in terms of the prevalence and levels of psychopathy in this type of population. For example, in the review of Beryl et al. (2014), a trend was found of lower rates of prevalence of psychopathy in European samples of women in prison or in prison hospitals than in American samples. Consistently, in the meta-analysis of Fox and DeLisi (2019), and after discarding the extreme values from samples composed exclusively of homicides with psychosis or psychopathy, significantly higher levels of PCL-measured psychopathy were found in homicide offenders from the USA and Canada than in homicide offenders from Finland, Sweden, and Germany.

Although these two reviews have reported that psychopathy prevalence is higher in North American male and female offenders and prisoners than in European male and female offenders and prisoners, the reasons for these differences are unclear. Beryl et al. (2014) suggest that the reason is “that the PCL instruments are designed to test the construct of ‘psychopathy’ as manifested in North American (male) offenders, and are less well-suited to identifying ‘psychopathy’ as manifested in European offenders” (p. 190). However, following the cultural facilitation model and Cooke et al.'s (2005) suggestions, an alternative reason is that complex social processes, such as socialization and enculturation, can suppress the development of certain aspects of psychopathy and facilitate the development of others. Therefore, it may be that socialization and enculturation in European countries suppress the development of certain psychopathic personality traits, or that those social processes in North American countries facilitate the development of certain psychopathic personality traits. There is also the possibility that both explanations are valid.

In any case, the results of the present review suggest that those differences between countries in the prevalence of psychopathy are unique to the prison or criminal population, but do not extend to the general population.

However, studies using samples from the general population of many different countries around the world have found cultural differences in the levels of different psychopathic traits. For example, in the study of Neumann et al. (2012) with 33,016 people (19,183 women) from 58 countries belonging to 11 world regions, significant differences were found between these regions in terms of the levels of different psychopathic traits (interpersonal, affective, antisocial, and lifestyle), as measured by one of the brief versions of the Hare SRP (SRP-E).

To further complicate the scenario of empirical results on the relationships between psychopathy and culture, the differences found in some studies with samples from the general population sometimes go in the opposite direction to those found in offender or prisoner populations. Thus, in the study of Lilienfeld et al. (2014), mentioned in the Introduction, in which they analyzed the responses of 3,338 people to the PPI-R-SF applied online, the Europeans showed higher levels of psychopathic traits than the Americans.

As a result, future research should address whether differences between countries in psychopathy only appear in terms of levels of certain psychopathic traits, but not in terms of the prevalence of psychopathy. When speaking about prevalence of psychopathy, we refer to it as defined by the presence of a clear set of psychopathic traits and with a certain level of intensity of such traits and/or a certain degree of impairment caused by such traits. It should also be examined whether such differences translate into a pattern of consistent differences between North American and European countries.

The results obtained in this work and the conclusions that have been reached should be assessed taking into account some of the limitations of the review itself. The most important limitations concern the high variability of the characteristics of the reviewed studies and the prevalence rates found, the small number of studies conducted to date that can help control such variability, and the methods assessing psychopathy in the reviewed studies. As already mentioned, prevalence rates vary greatly depending on factors such as the type of instrument used to define psychopathy, the participants' sex, and the type of sample from the general population. Given the small number of studies that currently constitute the scientific literature on the prevalence of psychopathy in the general population and the great heterogeneity of these studies in terms of their characteristics, it is very difficult to examine the effects of one of its factors while controlling the effect of the remaining factors. In fact, in this work, the number of subsamples/samples to examine gender prevalence was smaller than for calculating the overall prevalence. Therefore, in that smaller set, factors such as the type of instrument or sample did not reach statistical significance, thus preventing a more statistically potent analysis of the effect of gender after controlling the effects of these two factors and vice versa.

Among the factors that affected the variability of the prevalence of psychopathy, it is worth highlighting the type of instrument used to define psychopathy, since this factor points out important issue underlying this review. There is a high heterogeneity in the methods used to assess psychopathy in the reviewed studies. In addition, some of these method are more susceptible to criticisms related to their reliability and validity than others (e.g., the methods used in Hagnell et al., 1994; Gustafson and Ritzer, 1995; Pethman and Erlandsson, 2002). That heterogeneity and these criticisms go back to the problems in defining the construct of psychopathy. The different theoretical perspectives for this purpose which characterize the research of this construct are also an issue, and have already been discussed in the Introduction. In this sense, for example, an interesting exchange of views has recently been published on the debate over what components are essential to, or constitute part of psychopathy. It has also been discussed whether those components are necessary and/or sufficient (Brislin and Patrick, 2020a,b; Lynam, 2020; Marcus and Nagel, 2020). Consequently, one of the most important challenges that research in the area of psychopathy has to face is to achieve a valid and consensual definition of the construct of psychopathy and, related to this, to decide which instrument or instruments are the most valid and reliable to measure this construct. These needs are most evident when studying psychopathy in the general population because, as mentioned above, virtually all studies on psychopathy in the population of offenders or prisoners use the PCL-R or one of its versions (see the reviews of Beryl et al., 2014, and of Fox and DeLisi, 2019).

On the other hand, future research should also focus on the prevalence of the components of psychopathy, especially on the prevalence of psychopathic traits. Moreover, future research should also be conducted on the prevalence of the other personality constructs that are included under the Dark Triad label: Machiavellianism and narcissism.

Despite the above-mentioned limitations, the obtained results reflect relatively strong trends in the data that at least deserve to be the subject of future research and the formation of hypotheses to be taken into account in such research. In short, these trends allow the following conclusions to be drawn:

1. The prevalence of psychopathy in the general adult population can be estimated at 4.5%.
2. This prevalence is much lower than that found in the offender or prison population, which usually ranges between 10 and 35% (Nicholls et al., 2005; Guay et al., 2018; Fox and DeLisi, 2019).
3. The prevalence rates of psychopathy in the general population show considerable variation as a function of the type of instrument used to define psychopathy, the participants' sex, and the type of sample from the general population.
4. Using the PCL-R (or any of its versions), lower psychopathy prevalence rates are obtained than if self-reports of psychopathic personality traits are used.
5. As the PCL-R is currently considered the “gold standard” for the assessment and definition of psychopathy, the prevalence of psychopathy in the general population may be only 1.2% and, therefore, the difference with the prevalence of the offender or prison population may be even greater.
6. As is often the case in the offender and prison population, the prevalence of psychopathy in the general adult population is significantly higher among males than among females.
7. The prevalence of psychopathy is significantly higher among workers in some organizations and companies (e.g., managers, executives, procurement and supply professionals, advertising workers) than among university students or people from the general community. In turn, the prevalence of psychopathy among university students is significantly higher than among people from the general community, although the latter result could be due to the type of career that university students are pursuing (e.g., company careers vs. helping careers).

## Data Availability Statement

The raw data supporting the conclusions of this article will be made available by the authors, without undue reservation.

## Author's Note

This article is based in part on the final degree project carried out by AS-G under the direction of CG.

## Author Contributions

AS-G, CG, and JS contributed to conception and design of the study. AS-G organized the database and wrote the first draft of the manuscript. JS performed the statistical analysis. AS-G, JS, and MG-V wrote sections of the manuscript. All authors contributed to manuscript revision, read, and approved the submitted version.

## Conflict of Interest

The authors declare that the research was conducted in the absence of any commercial or financial relationships that could be construed as a potential conflict of interest.

## Publisher's Note

All claims expressed in this article are solely those of the authors and do not necessarily represent those of their affiliated organizations, or those of the publisher, the editors and the reviewers. Any product that may be evaluated in this article, or claim that may be made by its manufacturer, is not guaranteed or endorsed by the publisher.
